# Anticoccidial drugs of the livestock industry

**DOI:** 10.1007/s00436-019-06343-5

**Published:** 2019-05-31

**Authors:** Sandra Noack, H. David Chapman, Paul M. Selzer

**Affiliations:** 1 Boehringer Ingelheim Vetmedica GmbH, Ingelheim am Rhein, Germany; 2 Department of Poultry Science, University of Arkansas, Fayetteville, AR USA

**Keywords:** Coccidiosis, Anticoccidials, Livestock, Ionophores, Chemicals, Mode of action, Resistance

## Abstract

Coccidiosis is a parasitic disease of a wide variety of animals caused by coccidian protozoa. The coccidia are responsible for major economic losses of the livestock industry. For example, the annual cost due to coccidiosis to the global poultry industry has been estimated to exceed US$ 3 billion annually. Currently available drugs for the control of this disease are either polyether ionophorous antibiotics that are derived from fermentation products, or synthetic compounds, produced by chemical synthesis. Unfortunately, no new drugs in either category have been approved for use for decades. Resistance has been documented for all those of the drugs currently employed and therefore the discovery of novel drugs with unique modes of action is imperative if chemotherapy is to remain the principal means to control this disease. This chapter aims to give an overview of the efficacy and mode of action of the current compounds used to control coccidiosis in livestock and provides a brief outlook of research needs for the future.

## Introduction

Coccidiosis is an infectious disease of the intestinal tract of wild and domestic animals caused by different protozoa. These include but are not limited to *Isospora*, *Neospora*, *Cryptosporidium*, and *Eimeria* of the phylum *Apicomplexa.* Those parasites are widespread, especially where intensive production systems are used to raise livestock. They cause mortality, poor growth, and impaired performance. In addition to mortality and reduced growth, coccidiosis also affects meat yield and quality and increases susceptibility to ancillary infections (Nagi and Mathey 1972). In the chicken, at least seven *Eimeria* species are recognized that parasitize different regions of the intestine (Shirley et al. 1986); many species are recognized in ruminants. The parasites are transmitted from one host individual to others via the feces which shed the transmission stage of the life cycle (the oocyst) into the environment. Infection results from ingestion of sporulated oocysts in the litter. The global poultry industry is considered most affected by coccidiosis as it causes this industry annual losses that have been estimated to exceed US$ 3 billion per year (Williams 1999; Dalloul and Lillehoj 2006). Poultry production is projected to more than double by the year 2050 (Alexandratos and Bruinsma 2012) and control of coccidiosis will be essential if poultry and livestock meat is to fulfill the increasing need for protein by the growing world population (Kart and Bilgili 2008). Control of the disease by the use of drugs is indispensable if we are to achieve sustainable poultry production. Animal welfare is compromised by coccidial infections, and food safety is an important consideration (Kadykalo et al. 2018).

In addition to control programs based upon chemotherapy or vaccination, satisfactory control of coccidiosis in poultry requires strict attention to hygiene and sanitation, and biosecurity measures that limit human access to poultry facilities (Chapman 2018). Adequate ventilation and leak-free watering systems are important to reduce excessive moisture because wet litter aids sporulation of the infective stage of the life cycle (the oocyst). Nevertheless, despite such measures, eradication has not proved possible and the parasites persist in poultry flocks (Chapman et al. 2016). Preventative treatment may employ pharmaceutical ingredients in medicated food or drinking water, or immunization involving the use of live attenuated or non-attenuated vaccines (Klotz et al. 2005). By these means, it is estimated that most broiler chickens produced worldwide receive treatment with drugs or are vaccinated (Chapman et al. 2002). Prophylaxis has been the preferred method for the control of coccidiosis in poultry because treatment once clinical signs become apparent is often too late to prevent the pathological consequences of infection (Chapman 2009).

The concept of coccidiosis prevention in chickens by inclusion of drugs in the feed (prophylaxis) was first described in 1948 and involved the use of sulfaquinoxaline, the first feed additive for poultry (Grumbles et al. 1948; reviewed by Chapman 2009). In the years that followed, many other drugs were introduced, and until the introduction of ionophores in the 1970s, chemoprophylactic control of coccidiosis was based on the use of such synthetic anticoccidials (Ryley and Betts 1973). No new chemicals have been introduced for decades, and resistance has been documented for all the drugs approved for use in chickens (Chapman 1997), although the onset of resistance can be slowed by using rotation programs with different chemicals and/or ionophores (Chapman et al. 2010). Nevertheless, resistance to the available chemicals and ionophores has become widespread (Peek and Landman 2011). Drugs with novel molecular modes of action, and hence unprecedented targets, will be necessary if control of coccidiosis by chemotherapy is to be achievable in the future (Kinnaird et al. 2004; Scribner et al. 2009). Very little effort to discover new drugs has been undertaken in recent years, but this may change with the advent of genomics technology (Chapman et al. 2013). Examples of the successful application of novel drug discovery could be shown for other protozoa that are relevant for the animal health industry, e.g., for the pig parasite *Cystoiospora suis* (Shrestha et al. 2017a).

In this chapter, we aim to give an overview of the efficacy and mode of action of the current compounds used to control coccidiosis in livestock and provide a brief outlook of research needs for the future. Previous reviews of this subject include those by Chapman (1997) and McDougald (1982).

## Drug categories

Anticoccidial drugs belong to one of two categories (Chapman 1997; Allen and Fetterer 2002):

1. Polyether antibiotics or ionophores, which are produced by the fermentation of *Streptomyces* spp. or *Actinomadura* spp. These drugs disrupt ion gradients across the cell membrane of the parasite:

- Monovalent ionophores (monensin, narasin, salinomycin)
- Monovalent glycosidic ionophores (maduramicin, semduramicin)
- Divalent ionophore (lasalocid)

2. Synthetic compounds popularly known as “chemicals”, produced by chemical synthesis, often with a specific mode of action:

- Inhibition of parasite mitochondrial respiration (decoquinate, clopidol)
- Inhibition of the folic acid pathway (sulfonamides)
- Competitive inhibition of thiamine uptake (amprolium)
- Unknown mode of action (e.g., diclazuril, halofuginone, nicarbazin, robenidine)

Combination products, consisting of either a synthetic compound and ionophore (e.g., nicarbazin/narasin — Maxiban®, Elanco) or two synthetic compounds (clopidol/methyl benzoquate — Lerbek®, Impextraco NV), are also available. Arsenical drugs such as roxarsone that has some anticoccidial efficacy, arsanilic acid, carbarsone, and combinations thereof have been discontinued in many countries since 2015, based on scientific reports that indicated organic arsenic could transform into inorganic, highly toxic arsenic (Nachman et al. 2013; Huang et al. 2014).

### Ionophores

For many years, ionophores have been the principal choice to control coccidiosis because resistance develops slowly to them and because they do not completely suppress parasite development, thus allowing the development of immunity in the host after first exposure (Chapman 1999a, b; Chapman et al. 2010; Jeffers 1989). They are characterized by multiple tetrahydrofuran rings that are connected in the form of spiroketal moieties (Riddell 2002) and are effective against the asexual and sexual life cycle stages of coccidia, disturbing the normal transport of ions across surface membranes of sporozoites or early trophozoites (Augustine et al. 1992; Smith and Galloway 1983; Smith and Strout 1980; Smith et al. 1981). Ionophores are only used in livestock and are not employed for any purpose in human medicine. They are not active against most foodborne bacteria of poultry, e.g., *Escherichia coli, Salmonella* spp. and *Campylobacter* spp., and are not, therefore, included in the WHO list of medically important antimicrobials. Their use is not an issue for public health (Tang et al. 2017; WHO 2017).

These drugs have a rather narrow safety margin (Dowling 1992), and most are incompatible with several therapeutic antibiotics. Among those are tiamulin (Umemura et al. 1984a; Islam et al. 2009), chloramphenicol, erythromycin, oleandromycin (Umemura et al. 1984b; Mazlum et al. 1985; Mazlum and Pradella 1986; Perelman et al. 1986; Broz and Frigg 1987), and certain sulfonamides, leading to intoxication manifested by severe temporary clinical symptoms (Dowling 1992; Schuhmacher et al. 2006). In addition, ionophores are also incompatible with some antioxidants (Laczay 1988; Laczay et.al. 1988, 1989; Umemura et al. 1984b; Dowling 1992; Peek and Landman 2011).

Monovalent ionophores can form lipid-soluble complexes with sodium and potassium cations, whereas divalent ionophores can bind calcium and magnesium cations only. Polyether ionophores arrest the development of sporozoites by increasing the concentration of intracellular Na^+^ ions. In addition, they increase the activity of Na^+^/K^+^/ATPase (Wang et al. 2006) and affect merozoites by causing the cell membrane to burst (Mehlhorn et al. 1983). Toxic effects in horse, cattle, dogs, cats, rats, and avian species are thought to be mediated by disrupting ion gradients of cell membranes, leading to mitochondrial damage, and thus depletion of cellular energy. Well-known toxic effects are cardiac toxicity and muscle degeneration, and neuropathy, the latter one being manifested by myelin degeneration and ataxia (Chapman 2018; Kart and Bilgili 2008).

#### Monovalent ionophores

##### Monensin

**Fig. 1:**
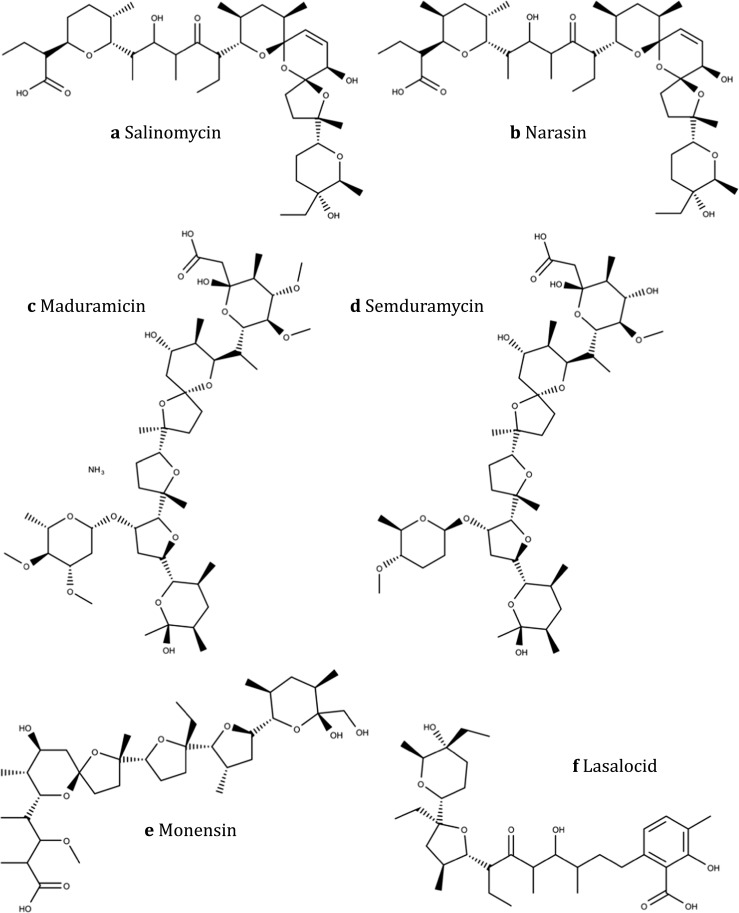
Ionophores used as anticoccidials. While salinomycin, narasin, maduramicin, semduramicin, and monensin (**a**–**e**) belong to the monovalent ionophores, lasalocid (**f**) is a divalent ionphore

In 1967, the structure of monensic acid (Fig. 1e), a fermentation product of *Streptomyces cinnamonensis*, was first described and the compound was reported to have a broad-spectrum effect against *Eimeria* (Agtarap et al. 1967). It forms lipid-soluble complexes with sodium and potassium cations, leading to increased permeability of the membrane for these ions. Monensin is able to transport sodium ions through membranes in both electrogenic and electroneutral manner (Mollenhauer et al. 1990). Horses are particularly susceptible to monensin poisoning (Matsuoka et al. 1996). Accidental deadly poisoning of horses with monensin has been published (Doonan et al. 1989; Bezerra et al. 1999; Bila et al. 2001).

##### Salinomycin

Salinomycin (Fig. 1a) was isolated from *Streptomyces albus*. It exhibits not only activity against *Eimeria* of poultry but also against gram-positive bacteria including mycobacteria and some filamentous fungi (Miyazaki et al. 1974). Salinomycin is an ionophore with strict selectivity for alkali ions and a strong preference for potassium, interfering with transmembrane potassium potential and promoting the efflux of K^+^ ions from mitochondria and cytoplasm. Recently, it has been shown to kill human cancer stem cells and to inhibit breast cancer growth and metastasis in mice (Naujokat et al. 2010). Salinomycin is the least toxic of all the ionophores (Oehme and Pickrell 1999).

##### Narasin

Narasin (Fig. 1b) is a polyether antibiotic obtained from *Streptomyces aureofaciens* (Jeffers et al. 1988). It is a derivative of salinomycin having an additional methyl group, therefore alternatively called (4S)-4-methyl salinomycin. When combining different ionophores with nicarbazin, Challey and Jeffers (1973) found that combinations of nicarbazin and narasin had synergistic activity. A combination product containing both active pharmaceutical ingredients (API) in 1:1 ratio was developed (Maxiban®). Very high levels of narasin caused death in sows, leg muscle weakness in turkeys, and cardiopulmonary clinical signs in 15% of the rabbits from Brazilian rabbit farms (Oehme and Pickrell 1999).

#### Monovalent glycosidic ionophores

##### Maduramicin

The ionophores maduramicin (also called Yumamycin; Fig. 1c) was first isolated from the bacterium *Actinomadura yumaensis* (Liu et al. 1983). It is a large heterocyclic compound with a series of electronegative crown ethers able to bind monovalent or divalent metal ions (Maron et al. 2015) and is widely used for commercial broiler production. Maduramicin is the most toxic of all the ionophores for non-target animals (Oehme and Pickrell 1999) and humans (Sharma et al. 2005). It might cause severe cardiovascular defects (Shlosberg et al. 1997), as it inhibits proliferation and induces apoptosis in myoblasts (Chen et al. 2014).

##### Semduramicin

Semduramicin (Fig. 1d) can be isolated from *Actinomadura roserufa* (Tynan et al. 1992). It is a highly effective drug against *Eimeria* and is well tolerated by chickens (Ricketts et al. 1992; Logan 1993).

#### Divalent ionophores

##### Lasalocid

Compound X-537A (later named lasalocid A; Fig. 1f) was isolated from *Streptomyces lasaliensis*. It was shown to have anticoccidial activity in chicken (Mitrovic and Schildknecht 1974) and to increase weight gain and feed conversion (Reid 1975).

With the exception of salinomycin, lasalocid is the one with lowest toxicity (Oehme and Pickrell 1999). Nevertheless, dogs appear to be more sensitive to lasalocid intoxication than other species, and accidental poisoning of dogs by lasalocid has been reported (Espino et al. 2003; Segev et al. 2004).

### Synthetic compounds

**Fig. 2:**
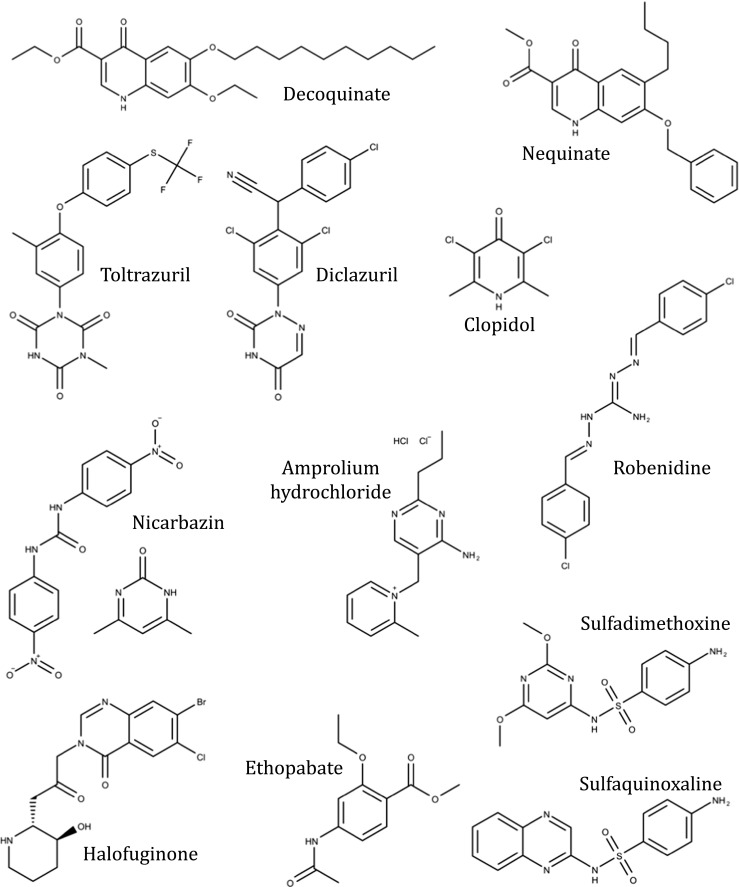
Synthetic anticoccidial APIs

Based on chemical structure, synthetic drugs include the quinolones, pyridones, alkaloids, guanidines, thiamine analogues, and triazine derivatives (Fig. 2). The mode of action of some synthetic anticoccidials has been described (Wang 1978, 1982) but for others their mode of action needs to be investigated (e.g., diclazuril, halofuginone, nicarbazin, robenidine). Such information and its relevance to the inhibition of specific developmental stages of the life cycle of the parasites are important in understanding toxicity and adverse effects of synthetic anticoccidials, and to obtain optimal control by correct timing of prophylaxis. Structures of synthetic anticoccidials are shown in Fig. 2.

#### Inhibition of parasite mitochondrial respiration

One of the main targets for anticoccidial drugs is the respiratory chain, which is different from vertebrates as *Eimeria tenella* oocysts predominantly use succinate or malate plus pyruvate to consume oxygen (Wang 1978). As the respiratory chain of protozoa is relatively insensitive to rotenone and amytal, it can be assumed that NADH dehydrogenase is less important than succinate dehydrogenase (Harder and Haberkorn 1989). Clopidol and quinolones inhibit mitochondrial energy production during the early stages of *Eimeria* development, but act on different strains of the coccidia (Anon 2010; Fry and Williams 1984).

##### Quinolones (decoquinate, nequinate (methyl benzoquate))

Quinolones were first discovered in 1962 and since then have undergone numerous modifications to their nucleus to improve spectrum as well as pharmacokinetics (Galarini et al. 2009). They arrest or kill sporozoites or early trophozoites, but even though they cover a broad spectrum, they are not able to fully control coccidiosis. Quinolone coccidiostats inhibit the respiration by blocking electron transport in the parasite mitochondrion reversibly, probably acting at a site near cytochrome b (Wang 1975, 1976).

Methyl benzoquate, an alkoxy-quinolone ester, acts synergistically with clopidol, a pyridone derivative to prevent *Eimeria* infections in chicken (Ryley 1975). Decoquinate (6-ethyl-(decycloxy)-7-ethoxy-4-hydroxy-3-quinolinecarboxylate) has been introduced in 1967 (Williams 2006). It also shows synergistic effects when combined with clopidol in low concentrations, inhibiting the electron transport more effectively than the sum of their individual actions (Fry and Williams 1984; Wang 1975; Williams 1997). Thus, both APIs are often used in combination products with clopidol. Nevertheless, quinolones and their combination with clopidol are used less frequently today because of the relatively rapid development of resistance (Chapman 1997; Kawazoe et al. 1991).

##### Clopidol

Clopidol, also known as meticlorpindol or clopindol, is a pyridinol with broad coccidiostatic activity against early development of *Eimeria* spp. by inhibiting mitochondrial energy production in sporozoites and trophozoites (Kant et al. 2013). A synergistic effect between meticlorpindol and 4-hydroxyquinolones has been described (Challey and Jeffers 1973; Jeffers and Challey 1973). To achieve complete control, combination products with quinolones are marketed.

##### Toltrazuril

Toltrazuril interacts with the mitochondrial pyrimidine biosynthesis linked to the respiratory chain; thus, it presumably inhibits mitochondrial dihydroorotate dehydrogenase (Harder and Haberkorn 1989; Jockel et al. 1998). In addition, toltrazuril might affect plastid-like organelles (Hackstein et al. 1995).

Toltrazuril is one of the triazines that acts on intracellular stages of the life cycle that are undergoing schizogony and gamogony (Haberkorn and Stoltefuss 1987), especially affecting mitochondria and the endoplasmatic reticulum (Mehlhorn et al. 1984). Respiratory chain enzymes like succinate-cytochrome C reductase, NADH oxidase and fumarate reductase as well as enzymes involved in pyrimidine synthesis are inhibited by toltrazuril. However, if one considers the high concentrations needed for inhibition of the latter enzymes, it is questionable whether this mechanism would translate into an anticoccidial effect (Harder and Haberkorn 1989). In addition, nuclear division in schizonts and microgamonts as well as the wall-forming bodies in macrogamonts are disturbed (Mehlhorn et al. 1984).

#### Inhibition of the folic acid pathway

The folic acid antagonists include sulfonamides, 2,4-diaminopyrimidines, and ethopabate, which are structural analogues of folic acid or of para-aminobenzoic acid (PABA), a precursor of folic acid. They interfere with the synthesis of folic acid by competing with PABA, thereby inhibiting folate synthetase, and thus preventing cellular replication (Lebkowska-Wieruszewska and Kowalski 2010). Diaveridine and ormetoprim are active against the protozoan enzyme dihydrofolate reductase (Lindsay et al. 1996). As coccidia rapidly synthesize nucleic acids, they have high requirements of folic acid — in contrast to their hosts, which are able to utilize folic acid from feed and thus, have no need for PABA (Zaionts et al. 1978).

##### Sulfonamides

Sulfonamides (sulfadimethoxine, sulfaquinoxaline) inhibit dihydropteroate synthetase (McCullough and Maren 1974). They have broad-spectrum activity against Gram-negative and Gram-positive bacteria as well as protozoa. Accidental human consumption of sulfonamide-contaminated products can cause central nervous system effects, gastrointestinal disturbances, and hypersensitivity reactions (Lebkowska-Wieruszewska and Kowalski 2010). Sulfonamides act on developing schizonts and on sexual stages.

Sulfonamides are only used very rarely in US broiler production because of the high potential for residues. On rare occasions only, a combination of sulfadimethoxine and ormetoprim is used in a “prestarter feed” for birds under 16 weeks of age to prevent mortality from coccidiosis and bacterial infections with a 5-day meat withdrawal period (United States Food and Drug Administration, FDA 2016). In Europe, sulfonamides are not approved for prevention of coccidiosis in poultry.

##### Ethopabate

Ethopabate is an antagonist of folic acid or of its precursor, PABA, thus inhibiting the synthesis of nucleic acid and limiting the production of new cells (Anon 2010). It is most active against *Eimeria maxima* and *Eimeria brunetti* (Peek and Landman 2011). As it lacks activity against *E. tenella* caecal stages, it is often used in combination products with amprolium.

#### Competitive inhibition of thiamine uptake

##### Amprolium

Amprolium hydrochloride (1-[(4-amino-2-propyl-5-pyrimidinyl)methyl]-2-methylpyridinium chloride monohydrochloride) is an analogue to thiamine (vitamin B_1_), but lacks the hydroxyethyl functionality that thiamine possesses and thus is not phosphorylated to a pyrophosphate analogue (Kart and Bilgili 2008). It inhibits the uptake of thiamine by second generation schizonts of *E. tenella* and prevents formation of thiamine pyrophosphate which is required for many essential metabolic reactions, e.g., as cofactor of several decarboxylase enzymes involved in cofactor synthesis (James 1980).

As amprolium is only poorly active against some *Eimeria* spp., it is largely used in combination products or mixtures with the folic acid antagonists ethopabate or sulfaquinoxaline to extend its spectrum of activity. The primary use of amprolium today is for water treatment during clinical outbreaks. Amprolium is the only active pharmaceutical ingredient approved for prevention and treatment in laying chicken. It has a large safety window (at least 5:1 when used at the recommended level in feed (125 ppm)) (Rychen et al. 2018).

#### Other modes of action

##### Nicarbazin

Nicarbazin is an equal molar complex of 4,4′-dinitrocarbanalide and 2-hydroxy-4,6-dimethylpyrimidine (Chapman 1994a). It was the first anticoccidial drug with a true broad-spectrum activity and has been in common use since 1955 (Anon 2010). The 4,4′-dinitrocarbanilide component of nicarbazin inhibits transglutaminase activity, whereas the 2-hydroxy-4,6-dimethylpyrimidine portion increases transglutaminase activity. In addition, nicarbazin increases lipoprotein lipase activity and acts as a calcium ionophore (Yoder et al. 2006). Nicarbazin and narasin show synergistic activity (Challey and Jeffers 1973) and a combination product of these drugs was developed. Nicarbazin has only a small safety window. As it disrupts the ion- and water equilibrium, medicated birds are at increased risk of heat stress under hot and humid weather conditions (Keshavarz and McDouglad 1981). In addition, it is highly toxic to layers — symptoms include bleaching of brown-shelled eggs, mottling of yolks, reduced hatchability, and decreased egg production (Jones et al. 1990).

##### Diclazuril

Like toltrazuril, diclazuril belongs to the chemical class of triazines, developed together with clazuril by Janssen Pharmaceuticals (Maes et al. 1988). Diclazuril is a nucleoside analogue thought to be involved in nucleic acid synthesis, possibly affecting later phases of coccidia differentiation (Verheyen et al. 1988). It has been shown to affect parasite wall synthesis resulting in the formation of an abnormally thickened, incomplete oocyst wall, and zygote necrosis in both *E. brunetti* and *E. maxima* (Verheyen et al. 1989). In addition, diclazuril has been shown to cause disruption of transmembrane potential of mitochondria and to induce ultrastructural changes in merozoites (Zhou et al. 2010). Nevertheless, it is not clear if this is the true mode of action or is just a consequence of cell death. Diclazuril was shown to downregulate mRNA expression of the serine/threonine protein phosphatase type 5 (PP5) significantly by 51.4% in *E. tenella* (Zhou et al. 2013). PP5s of many eukaryotic organisms have important regulatory functions in the cell cycle (Dobson et al. 2001; Lindenthal and Klinkert 2002) and are associated with the apoptosis signal-regulated kinase 1 (ASK1) (Kutuzov et al. 2005).

#### Unknown modes of action

##### Halofuginone

Halofuginone hydrobromide is a quinazolinone derivative related to the antimalarial drug febrifuginone. It was originally extracted from leafs and roots of the traditional Chinese herbal *Cichroa febrifuga* plant, which is used traditionally in Chinese medicine to treat malaria (Pines et al. 2000). It is effective against asexual stages of most species of *Eimeria*, delaying development (Zhang et al. 2012).

##### Robenidine

Robenidine (1-3-bis (p-chlorobenzylideneamino)-guanidine hydrochloride) is a synthetic derivative of guanidine introduced in 1972 (Kennett et al. 1974), which does not affect initial intracellular development of coccidian, but prevents formation of mature schizonts. Its MoA is presumed to interfere with energy metabolism by inhibition of respiratory chain phosphorylation and ATPases, and to inhibit oxidative phosphorylation (Wong et al. 1972; Kant et al. 2013).

## Markets and market products

**Fig. 3:**
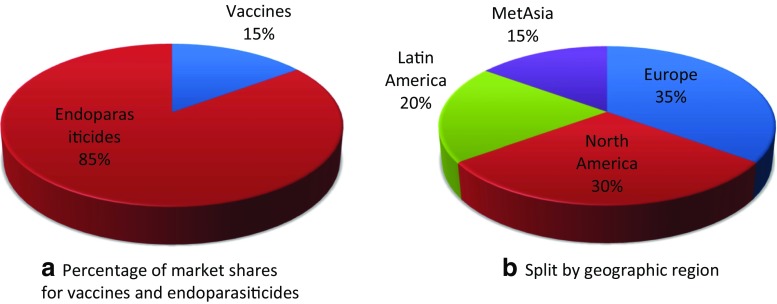
The global anticoccidial poultry market. **a** Endoparasiticides make up 85% of the total global anticoccidial poultry market; **b** the North American and European poultry market are currently the most relevant ones (Boehringer Ingelheim internal analysis 2016)

About 59 billion broilers, 5.8 billion layers, and 1400 billion eggs are produced each year worldwide. The global anticoccidial poultry market as estimated by the animal health industry is approx. US$ 1 billion (based on Boehringer Ingelheim internal analysis 2016). Academic estimates calculate that the global loss due to coccidiosis in poultry is in excess of US$ 3 billion (Williams 1999; Shirley et al. 2007). Specifying a value of the market and the cost of coccidiosis in poultry is not an easy if not an impossible task because small errors in such calculations can cause huge differences and there are many different estimates and continuously changing factors involved (e.g., currencies, inflation, energy cost) (Williams 1999). Nevertheless, it is reasonable to speculate how much of these costs can be exploited commercially. If only half of the estimates, it would still be in the range of US$ 0.5 billion and therefore deserving of further research. To illustrate distinctions in differently regulated markets, we will focus in the next sections on marketed products in Europe, the USA, and Australia (Fig. 3).

### European Union

In the EU, chemicals are rarely used apart from the synthetic/ionophore combination product Maxiban®. In contrast, in the USA synthetic anticoccidials are often employed in rotational programs with ionophores. Nevertheless, they represent a minor part of the coccidiosis control program. Ionophores are most widely used in the EU, also due to their antibiotic affects in the intestine, e.g., against dysbacteriosis caused by clostridia. The ionophores salinomycin, narasin, monensin, lasalocid, maduramicin, and semduramicin and the chemical anticoccidial drugs robenidine, decoquinate, halofuginone, nicarbazin, and diclazuril are licensed in the EU as zootechnical feed additives under regulation 1831/2003/EC in species where coccidiosis is *systematic* for biological and zootechnical reasons, which is the case for poultry and rabbits. Systematic means that in these species, diagnosis of coccidiosis is not required and therefore, no prescription is necessary. By contrast, in species where coccidiosis is *not systematic*, anticoccidials are registered as veterinary medicines (e.g., for cattle; regulated in the European Union by Directive 2001/82/EC). In the UK, more than 40% of all antimicrobials sold for use in food and non-food animals are employed for the control of coccidia (277 tons of active ingredient in 2011; mostly for control of *Eimeria*) with ionophores representing more than 70% of these (Veterinary Medicines Directorate 2012) (Table 1).

**Table 1:** Anticoccidial products and APIs approved in Europe for use in poultry (data retrieved from European Food Safety Authority http://www.efsa.europa.eu/, Department for Environment, Food & Rural Affairs https://www.vmd.defra.gov.uk/ProductInformationDatabase)

Anticoccidial API	Trade name	Company
Decoquinate	Deccox	Alpharma BVBA; Zoetis SA
Diclazuril	Clinacox Coxiril	Eli Lilly and Company Ltd; Janssen Pharmaceutica NV Huvepharma NV
Halofuginone	Stenorol	Huvepharma NV
Lasalocid A	Avatec	Alpharma BVBA; Zoetis SA
Maduramicin	Cygro	Alpharma BVBA; Zoetis SA
Monensin	Coxidin	Huvepharma NV
	Elancoban	Eli Lilly and Company Ltd
Narasin	Monteban	Eli Lilly and Company Ltd
Narasin + nicarbazin	Maxiban	Eli Lilly and Company Ltd
Nicarbazin	Koffogran (Nicarb)	Phibro Animal Health SA
Robenidine	Robenz (Cycostst)	Alpharma BVBA; Zoetis SA
Salinomycin	Huvesal, Sacox	Huvepharma EOOD; Huvepharma NV
	Salinomax	Alpharma BVBA
Semduramicin	Aviax 5%	Phibro Animal Health SA
Toltrazuril	Baycox	Bayer AH

### USA

Rotation programs including ionophores and synthetic anticoccidials are the standard in intensive broiler production in the USA. These programs sometimes are also combined with vaccination. In contrast to the EU, the use of antibacterials for growth promotion is allowed in the USA. However, with the Guidance for Industry (GFI) 209 and GFI 213 (FDA 2013), the FDA has enhanced control of use of medically important antibacterials, eliminating the use of them for growth promotion. In addition, in 2017, the USA restricted the use of medically important antibiotics in feed to Veterinary Feed Directives (VFD) that require veterinary oversight (Castanon 2007; Federal Register 2015). The use of a VFD drug in feed is permitted only under the professional supervision of a licensed veterinarian (https://www.fda.gov/animalveterinary/developmentapprovalprocess/ucm455416.htm), while administration in drinking water still requires prescription. This restriction led to the withdrawal of some old anticoccidials, as for all, new VFD registrations were required, especially for combination products (Table 2).

**Table 2:** Anticoccidial products and APIs approved by the FDA for use in poultry (data retrieved from U.S. Food & Drug Administration https://www.fda.gov/AnimalVeterinary/default.htm)

Anticoccidial API	first approved	Trade names	Company	Combination products available with
Amprolium	1960	Amprol; Corid; Amprolium-P	Huvepharma EOOD	Bacitracin,
		AmproMed P	Cross Vetpharm Group Ltd.	Bambermycins,
		Cocciprol	Phibro Animal Health Corp.	Virginiamycin
Clopidol	1968	Coyden 25	Huvepharma EOOD	Bacitracin, Bambermycins, Chlortetracycline
Decoquinate	1970	Deccox	Zoetis Inc.	Bacitracin, Chlortetracycline, Lincomycin
Diclazuril	1999	Clinacox	Huvepharma EOOD	Bacitracin, Bambermycins, Virginiamycin
Halofuginone	1987	Stenorol	Huvepharma EOOD	Bacitracin, Bambermycins
Lasalocid	1976	Avatec	Zoetis Inc.	Bacitracin, Bambermycins, Virginiamycin
Maduramicin	1989	Cygro	Zoetis Inc.	–
Monensin	1971	Coban 90 Coban 60	Elanco US Inc.	Avilamycin, Bacitracin, Bambermycins, Chlortetracycline, Lincomycin, Ractopamine, Oxytetracycline, Virginiamycin, Tilmicosin
Narasin	1988	Monteban 45	Elanco US Inc.	Avilamycin, Bacitracin, Bambermycins
Nicarbazin	1955	Nicarb 25%	Phibro Animal Health Corp.	Bacitracin, Bambermycins
		Nicarbazin; Carbigran 25	Elanco US Inc.	
		Nicarmix 25	Planalquimica Industrial Ltda.	
Robenidine	1972	Robenz	Zoetis Inc.	Bacitracin, Chlortetracycline, Lincomycin, Oxytetracycline
Salinomycin	1983	Bio-Cox Type A Medicated Article Sacox 60	Huvepharma EOOD	Avilamycin, Bacitracin, Bambermycins, Chlortetracycline, Lincomycin, Oxytetracycline, Virginiamycin
Semduramicin	1995	Aviax	Phibro Animal Health Corp.	Bacitracin, Virginiamycin
Sulfachloropyrazine		ESB 3	Zoetis Inc.	–
Sulfamethazine	1945	SMZ-Med 454 Sulmet Soluble Powder	Cross Vetpharm Group Ltd. Huvepharma EOOD	–
Sulfadimethoxine		ALBON; AGRIBON SDM Sulfadimethoxine Concentrated Solution 12.5% Sulfadimethoxine Soluble Powder Sulfamed-G Sulfasol Soluble Powder; Sulforal DI-METHOX; Sulfadimethoxine 12.5% Oral Solution; Sulmet Drinking Water Solution, 12.5%	Zoetis Inc. Cronus Pharma LLC Phibro Animal Health Corp. Cross Vetpharm Group Ltd. Med-Pharmex, Inc. Huvepharma EOOD	–
Sulfaquinoxaline	1948	20% Sulfaquinoxaline Sodium Solution; 25% S.Q. Soluble; S.Q. 40%; Sul-Q-Nox Sulquin 6-50 Concentrate	Huvepharma EOOD Zoetis Inc.	–
Zoalene	1960	Zoalene 90 Medicated Coccidiostat Zoamix Type A Medicated Article	Zoetis Inc.	Bacitracin, Bambermycins, Lincomycin
Amprolium + ethopabate	1997	Amprol Plus Amprol Hi-E Amprol Plus 3-Nitro	Huvepharma EOOD	Bacitracin, Bambermycins, Chlortetracycline
Narasin + nicarbazin	1989	Maxiban 72	Elanco US Inc.	Avilamycin, Bacitracin, Bambermycins
Ormetoprim + sulfadimethoxine	1970	Rofenaid 40	Zoetis Inc.	–
Sulfamethazine + sulfaquinoxaline	2006	PoultrySulfa	Huvepharma EOOD	Sulfamerazine

### Australia

All agricultural and veterinary chemical products sold in Australia have to be registered by the Australian Pesticides and Veterinary Medicines Authority (APVMA). The “Guideline for the evaluation of the efficacy and safety of coccidiostats” (https://apvma.gov.au/node/427) has to be followed (Table 3).

**Table 3:** Anticoccidial products and APIs approved in Australia for use in poultry (data retrieved from Australian Pesticides and Veterinary Medicines Authority https://apvma.gov.au)

Anticoccidial API	First approved	Trade names	Company
3,5-Dinitro-O-toluamide	1994	Dot	Dox-Al Australia PTY Ltd
		Dot premix	Bec Feed Solutions PTY Ltd
		Doteco	International Animal Health Products PTY Ltd
		Nutridot	Nutriment Health PTY Ltd
		Phibrodot	Phibro Animal Health PTY Limited
Amprolium	1996	Amprolium	Parafarm PTY Ltd
Decoquinate	2016	Deccox	Zoetis Australia PTY Ltd
Lasalocid	2001	Avatec	Zoetis Australia PTY Ltd
Maduramicin	1997	CyGro	Zoetis Australia PTY Ltd
		Maduradox	Dox-Al Australia PTY Ltd
Monensin	1994	CCD Monensin, Rumensin	Elanco Australasia PTY Ltd
		Coxidin	Huvepharma EOOD
		Doxaban	Dox-Al Italia S.P.A.
		Monendox	Dox-Al Australia PTY Ltd
		Moneco	International Animal Health Products PTY Ltd
		Neove Monensin	Nutriment Health PTY Ltd
		Phibromonensin	Phibro Animal Health PTY Limited
Narasin	1984	Elanco Narasin, Monteban	Elanco Australasia PTY Ltd
Nicarbazin	1996	Carbidox	Dox-Al Australia PTY Ltd
		Cycarb	Zoetis Australia PTY Ltd
		Elanco Nicarbazin, Carbigran	Elanco Australasia PTY Ltd
		Keymix	International Animal Health Products PTY Ltd
		Nutrinicarb	Nutriment Health PTY Ltd
		Phicarb	Phibro Animal health PTY limited
Robenidine	2003	Cycostat	Zoetis Australia PTY Ltd
		Nutrirob	Nutriment Health PTY Ltd
Salinomycin	1996	Bio-Cox, Sacox	Huvepharma EOOD
		CCD Salinomycin	CCD Animal Health PTY Ltd
		Coxistac	Phibro Animal Health PTY Limited
		Neove	Nutriment Health PTY Ltd
		Sadox, Salindox	Dox-Al Australia PTY Ltd
		Doxalino	Dox-Al Italia S.P.A
		Saleco	International Animal Health Products PTY Ltd
Semduramicin	1998	Aviax	Phibro Animal health PTY limited
Sulfaquinoxaline	1983	Inca Sulpha-Quin	Inca (Flight) Co PTY Ltd
Toltrazuril	1993	Baycox, Toltracox Poultry	Bayer Australia Ltd. (Animal Health)
		Coxi-Stop	Abbey Laboratories PTY Ltd
Diaveridine + sulfaquinoxaline	1990	Keymix Solquin	International Animal Health Products PTY Ltd
Amprolium + ethopabate	1989	Keymix Keystat	International Animal Health Products PTY Ltd
Maduramicin + nicarbazin	2005	Gromax	Zoetis Australia PTY Ltd
Methyl benzoquate + clopidol	1993	Lerbek	Feedworks PTY Ltd

## Control of coccidiosis in ruminants and swine

As with poultry, *Eimeria* infections in ruminants are ubiquitously present in the environment and occur wherever animals are raised, especially in heavily stocked pasture with intensive grazing and crowded conditions such as feedlots (Daugschies and Najdrowski 2005; Keeton and Navarre 2018; Taylor and Catchpole 1994). In a study on first-year grazing cattle in Germany, up to 90% of all animals showed *Eimeria*-positive fecal samples (von Samson-Himmelstjerna et al. 2006). Probing the scientific literature, it seems that only little research in ruminants has been undertaken (or at least published) for many years, and only a few drugs are currently approved for use. Many of the difficulties in the interpretation of drug efficacy have been described (Gregory et al. 1982). For example, a decrease in oocyst production may follow treatment but this may not be associated with better weight gain and clinical condition. Many field studies show the effects of drugs upon oocyst production but effects upon clinical criteria require artificial inoculation of very large numbers of oocysts. To provide a framework for evaluation of efficacy of anticoccidials in mammals, a respective WAAVP guideline has been published recently (Joachim et al. 2018). This guideline proposes how to conduct both experimental and field studies for dose determination, dose confirmation, and assessment of field effectiveness to obtain solid efficacy data. In addition, guidance on selection of animals, diagnostic techniques, statistical evaluation, and methods concerning preparation, maintenance, and application of parasites is provided.

There are several anticoccidial drugs available for treatment and prevention of coccidiosis in ruminants, both from the class of synthetic drugs (e.g., sulfonamides, amprolium, decoquinate, the triazines diclazuril and toltrazuril) and ionophores (monensin, lasalocid). These drugs are used either therapeutically for the treatment of young animals showing signs of infection (e.g., amprolium, sulfonamides; triazines) or for prevention by inclusion in the feed (e.g., decoquinate, monensin, lasalocid). As most of the damage to the intestine is already present before diagnosis of coccidiosis occurs, therapy is mostly not sufficient to cure, but rather useful to hinder further spread of disease. Therapy has to be combined with palliative application of electrolytes, glucose, and anti-diarrheals to help to maintain hemostasis (Daugschies and Najdrowski 2005).

Only three APIs are currently used for treatment of porcine coccidiosis: narasin, salinomycin, and toltrazuril. Resistance against toltrazuril has been observed not only for *Eimeria* isolates from chicken (Stephan et al. 1997) and ovine *Eimeria* field isolates (Odden et al. 2018), but also in a field isolate of *Cystoisospora suis*, the usual cause of coccidiosis in very young pigs (Shrestha et al. 2017b).

### Ionophores

The divalent ionophore lasalocid has been shown to decrease oocyst production in naturally infected lambs and cattle and in a few cases improve performance and reduce clinical signs of disease when included in the feed (Foreyt et al. 1986; Horton and Stockdale 1981). The efficacy of the monovalent ionophore monensin, employed as a preventive drug by incorporation in the feed of livestock, is well documented (e.g., Bergstrom and Maki 1976; Fitzgerald and Mansfield 1978; Gregory et al. 1983; Leek et al. 1976; McDougald 1978).

### Amprolium

Amprolium has been used for many years for the control coccidiosis in sheep and cattle. The drug is principally used for the treatment of animals showing clinical signs of disease but may be employed for prevention by inclusion in the feed. It is available as an oral solution, soluble powder, or as a pelleted feed additive. The drug was shown to reduce oocyst production in lambs when given as an in-feed medication and an outbreak of clinical coccidiosis was successfully controlled by single drenching followed by medication (Talmon et al. 1989). Amprolium was effective for the control of coccidiosis in feedlot lambs (Baker et al. 1972) and in cattle (Norcross et al. 1974).

### Decoquinate

This drug is used for the prevention of coccidiosis in young ruminants by incorporation in the feed. Its efficacy has been demonstrated in sheep, goats, and cattle (Fitzgerald and Mansfield 1989; Foreyt 1987; Miner and Jensen 1976).

### Triazines

Numerous studies have shown that diclazuril and toltrazuril, when administered orally to young cattle, lambs, or pigs prior to the onset of clinical signs (referred to as metaphylactic treatment), decreases oocyst production in natural and artificial infections with *Eimeria* species (Bohrmann 1991; Mundt et al. 2003a, 2005, 2009; Epe et al. 2005; Daugschies et al. 2007) or *Isospora suis* (Driesen et al. 1995; Mundt et al. 2003b, 2007; Kreiner et al. 2011). In some cases, improvements in performance have been demonstrated (e.g., Ruiz et al. 2012; Scala et al. 2009; Rypula et al. 2012).

### Sulfonamides

Various sulfonamides, such as sulfaquinoxaline and sulfaguanidine, have been used for many years for the treatment of livestock showing clinical signs of coccidiosis (e.g. Hammond et al. 1956). As appetite is depressed in infected animals then inclusion in the drinking water is preferable for treatment (Shumard 1957).

### Registered APIs in different markets

#### European Union

In the European Union, only few anticoccidial APIs are registered for use in ruminants and/or pigs (mostly piglets): decoquinate, diclazuril, lasalocid, monensin, and toltrazuril (European Medicines Agency www.EMA.Europa.eu).

#### USA

In the USA, anticoccidials for use in ruminants and pigs are mainly used in combinations as feed additives, combined with antibacterials and growth promoters. Registered APIs are as follows:

**Table:** 

Amprolium	Clopidol	Decoquinate	Dexamethasone
Diclazuril	Ethopabate	Halofuginone	Lasalocid
Maduramicin	Monensin	Narasin	Nicarbazin
Ormetoprim	Robenidine	Salinomycin	Semduramicin
Sulfachloropyrazine	Sulfadimethoxine	Sulfamerazine	Sulfamethazine
Sulfaquinoxaline	Tylosin	Zoalene	

(Source: U.S. Food & Drug Administration https://www.fda.gov/AnimalVeterinary/default.htm).

#### Australia

In Australia, only lasalocid, monensin, narasin, salinomycin, and toltrazuril are registered anticoccidials for treatment of ruminants and/or pigs (Australian Pesticides and Veterinary Medicines Authority https://apvma.gov.au).

## Resistance and research for new anticoccidials

Development of resistance is a threat for all drugs that are used extensively for a prolonged time. A consequence of this is documented resistance for all drugs in intensive poultry production (Table 4) (Chapman 1997). In a survey conducted in the USA from 1995 to 1999, it was found that anticoccidials were universally used by 99% of commercial broiler operations (Chapman 2001).

**Table 4:** Summary of reported resistance to anticoccidials in field strains of *Eimeria* (adapted from Chapman 1997)

Drug	Year of introduction	Country where resistance was first described	Year resistance was first described	Species^a^
Sulphaquinoxaline	1948	USA	1954	*Et*
Nitrofurazone	1948	USA	1955	Not given
Nicarbazin	1955	Britain	1964	*Et*
Dinitolmide	1960	Britain	1964	*Et*, *En*
Amprolium	1960	Britain	1964	*Eb*
Clopidol	1966	Britain	1969	*Ea*, *Em*, *Et*
Buquinolate	1967	USA	1968	Not given
Methyl benzoquate	1967	Britain	1970	*Et*
Decoquinate	1967	Britain	1970	*Et*
Monensin	1971	USA	1974	*Em*
Robenidine	1972	USA	1974	*Em*
Halofuginone	1975	France	1986	*Ea*, *Et*
Lasalocid	1976	USA	1977	*Ea*
Arprinocid	1980	Britain	1982	*Et*
Salinomycin	1983	USA	1984	Various
Diclazuril	1990	Brazil	1994	*Ea*, *Em*, *Et*
Toltrazuril	1986	Netherlands	1993	Not given

^a^Species: *Ea*, *E. acervulina*; *Eb*, *E*. *brunetti*; *Em*, *E. maxima*; *En*, *E*. *necatrix*; *Et*, *E. tenella*

**Fig. 4:**
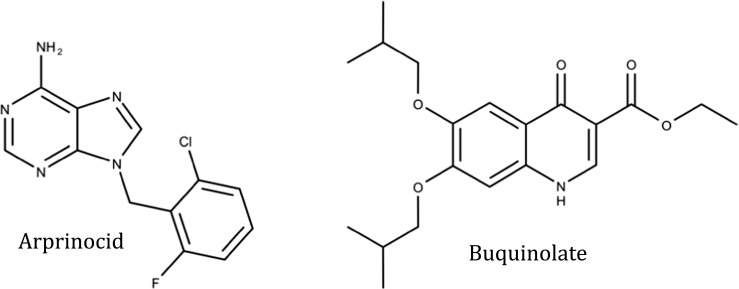
Chemical structures of arprinocid and buquinolate

The polyether ionophores became the drug of choice in 1972 and remain the most extensively used drugs in poultry as of today. While the development of resistance to ionophores is rather slow probably due to their unique mode of action, resistance development in synthetic drugs that have a specific mode of action seems to appear more rapidly, involving genetic mechanisms. Sometimes resistance was reported shortly after marketing a new drug, an example being arprinocid (Fig. 4) (Chapman 1983). Another example is the quinolone buquinolate (Fig. 4) that was “commercially dead” within 6 months of its introduction due to the sudden appearance of drug resistance. Development of resistance against buquinolate was found to take place after a single experimental passage of *Eimeria* (Chapman 1975). By comparison, drug resistance against toltrazuril did not occur in at least five successive drug exposures in field studies (Claeskens et al. 2007).

A study on ten *Eimeria* field isolates from northern Germany detected resistance against in nine out of the ten isolates of *Eimeria*, with seven out of the ten having developed multiple resistance (Stephan et al. 1997). Similar results have been shown in isolates from broiler farms in the UK (Chapman 1993) and the Netherlands (Peek and Landman 2006), showing the enormous threat of development of broad resistance against all classes of anticoccidial drugs. As drug sensitivity in a population of coccidia can be altered by the introduction of drug-sensitive coccidia, e.g., through the use of coccidiosis vaccines, or by the use of drug-sensitive laboratory-maintained lines or other reservoirs (Ball 1966; Jeffers 1976; McLoughlin and Chute 1978), these measures have to be combined in an attempt to control coccidiosis. Restoration of sensitivity to drugs following the use of vaccines comprising drug-sensitive strains of *Eimeria* has been demonstrated for the ionophores, monensin, and salinomycin, and the synthetic drug diclazuril (Chapman 1994b; Chapman and Jeffers 2014, 2015; Jenkins et al. 2010; Peek and Landman 2006; Mathis and Broussard 2006). Partial restoration of sensitivity to diclazuril and monensin was also observed following use of attenuated vaccine (Peek and Landman 2006). Therefore, a yearly rotation program has been proposed in which use of ionophores is alternated in successive flocks with vaccination (Chapman et al. 2010).

The development of resistance led to increasing efforts for the identification of new, resistance-breaking drugs. One recent example is nitromezuril, a new triazine anticoccidial. It shows only limited cross-resistance with diclazuril or toltrazuril (Fei et al. 2013), but these results have to be further evaluated before this drug might finally enter the market.

## Conclusion

In the light of the continuously expanding livestock industry and its growing significance for global food production, control of coccidiosis, perhaps the most widespread and intractable disease of poultry and other livestock, is of great importance; however, relatively little effort has been made on *Eimeria* infections of domestic livestock (Engels et al. 2010; Fernandez et al. 2012; Marhöfer et al. 2013; Müller and Hemphill 2016). In view of advances in biotechnology, modern approaches towards the discovery of novel resistance-breaking drug candidates may be anticipated. Genomic analysis of all seven *Eimeria* species that cause coccidiosis in poultry has been accomplished (Reid et al. 2014) and this may allow the identification and validation of species-specific protein targets. Novel drug discovery rationales including high throughput screening, structural biology, and the elucidation of the mode of action of active compounds can be envisioned. Combining target-based approaches with parasite in vitro and in vivo testing and medicinal chemistry generates a comprehensive view on the genotype-to-phenotype-to-compound correlation, which could allow for the design of novel drug candidates. Unfortunately, resistance develops rapidly following the introduction of drugs in the field. The current approach to delay the onset of resistance is to employ rotation programs combined with good husbandry, chemoprophylaxis, and/or live parasite vaccination (Blake et al. 2017). However, for the control of coccidiosis in the future, both novel cost-effective preventative chemotherapy and subunit or recombinant vaccines are desperately needed.
